# Sleep and Wake Affect Glycogen Content and Turnover at Perisynaptic Astrocytic Processes

**DOI:** 10.3389/fncel.2018.00308

**Published:** 2018-09-11

**Authors:** Michele Bellesi, Luisa de Vivo, Samuel Koebe, Giulio Tononi, Chiara Cirelli

**Affiliations:** ^1^Department of Psychiatry, University of Wisconsin-Madison, Madison, WI, United States; ^2^Department of Experimental and Clinical Medicine, Section of Neuroscience and Cell Biology, Università Politecnica delle Marche, Ancona, Italy

**Keywords:** mouse, cerebral cortex, sleep, sleep deprivation, chronic sleep restriction, glycogen

## Abstract

Astrocytic glycogen represents the only form of glucose storage in the brain, and one of the outcomes of its breakdown is the production of lactate that can be used by neurons as an alternative energetic substrate. Since brain metabolism is higher in wake than in sleep, it was hypothesized that glycogen stores are depleted during wake and replenished during sleep. Furthermore, it was proposed that glycogen depletion leads to the progressive increase in adenosine levels during wake, providing a homeostatic signal that reflects the buildup of sleep pressure. However, previous studies that measured glycogen dynamics across the sleep/wake cycle obtained inconsistent results, and only measured glycogen in whole tissue. Since most energy in the brain is used to sustain synaptic activity, here we employed tridimensional electron microscopy to quantify glycogen content in the astrocytic processes surrounding the synapse. We studied axon-spine synapses in the frontal cortex of young mice after ~7 h of sleep, 7–8 h of spontaneous or forced wake, or 4.5 days of sleep restriction. Relative to sleep, all wake conditions increased the number of glycogen granules around the synapses to a similar extent. However, progressively longer periods of wake were associated with progressively smaller glycogen granules, suggesting increased turnover. Despite the increased number of granules, in all wake conditions the estimated amount of glucose within the granules was lower than in sleep, indicating that sleep may favor glucose storage. Finally, chronic sleep restriction moved glycogen granules closer to the synaptic cleft. Thus, both short and long wake lead to increased glycogen turnover around cortical synapses, whereas sleep promotes glycogen accumulation.

## Introduction

The human brain has remarkably high energy demands, accounting for ~20% of the body metabolism (Attwell and Laughlin, 2001). Glucose is the main energetic substrate of the brain, and increases in local blood flow and glucose utilization typically follow changes in brain activity (Bélanger et al., 2011).

While local increases in cerebral blood flow are well recognized phenomena, the cellular responses to energy demand remain controversial. Nerve endings can directly uptake and oxidize glucose under both resting and activated conditions (Patel et al., 2014). Consistent with these findings, a recent study used two-photon imaging of a near-infrared 2-deoxyglucose analog to estimate cellular glucose uptake, observing that glucose is taken up preferentially by neurons in awake mice and, following sensory stimulation, in anesthetized mice (Lundgaard et al., 2015). Using genetically-encoded fluorescent biosensors, another study assessed the metabolic responses of individual neurons to stimulation and found increased direct glucose consumption by neurons, thus confirming that neurons can directly utilize glucose as needed (Díaz-García et al., 2017). Other lines of evidence support the “astrocyte-neuron lactate shuttle” (ANLS) hypothesis (Pellerin and Magistretti, 1994; Barros et al., 2009; Chuquet et al., 2010; Magistretti and Allaman, 2018), which proposes that astrocytes primarily uptake glucose from the blood, and upon glutamatergic stimulation, glucose is converted to lactate in astrocytes, which can be then extruded into the extracellular space. The released lactate can be used by neurons as an additional oxidative substrate to satisfy their energy needs (Pellerin and Magistretti, 1994; Pellerin et al., 2007; Bélanger et al., 2011) and to promote synaptic plasticity (Suzuki et al., 2011; Yang et al., 2014; Magistretti and Allaman, 2018). The exclusive compartmentalization of glycogen in astrocytes, together with the robust production of lactate upon glycogenolysis, potentially enables the astrocytes to buffer sudden increases in energy requirements (Bouzier-Sore and Pellerin, 2013).

In astrocytes glucose is stored in the form of glycogen, which represents the only long-term glucose deposit in the brain (Obel et al., 2012). During neuronal activation, glycogen is quickly mobilized to glycolytically produce lactate (Dringen et al., 1993; Walls et al., 2009) or, alternatively, it can promote glucose uptake from the *interstitium* to nearby activated neurons (DiNuzzo et al., 2010). Sustained neuronal activity can lead to the depletion of glycogen stores in the brain. This finding, together with the fact that overall neuronal activity often decreases during sleep, has led to the hypothesis that one of the functions of sleep is to replenish glycogen stores that are progressively depleted during wake (Benington and Heller, 1995; Scharf et al., 2008). Thus, many attempts have been carried out to measure changes in glycogen content between sleep and wake. However, although there is substantial evidence showing increased expression of key transcripts involved in glycogen synthesis during wake (Petit et al., 2002, 2010; Bellesi et al., 2015), direct measurements of glycogen amount across the sleep/wake cycle have led to inconsistent results (Gip et al., 2002; Kong et al., 2002; Franken et al., 2003, 2006; Petit et al., 2010). Early experiments using rapid brain freezing found a large increase of cerebral glycogen content during sleep, which dissipated very quickly upon awakening (Karnovsky et al., 1983). More recently, high-energy focused microwave irradiation was used to inactivate enzymes implicated in the metabolism of glycogen (Gip et al., 2002). With this method glycogen levels were found to decrease in the cerebellum of young rats after 6 h of sleep deprivation (SD), increase in the cerebral cortex of adult rats (p59) after 12 h of SD (Gip et al., 2002), and decrease in the rat forebrain after 12 or 24 h of SD (Kong et al., 2002). With the same microwave irradiation method levels of glycogen were found to increase in the cerebral cortex of B6 mice but did not change in AK and D2 mice, while in the latter two strains they decreased in brainstem and cerebellum (Franken et al., 2003, 2006). No change in glycogen levels were found in OF1 outbred mice after periods of wake enforced either by exposing the mice to novel objects and new nesting material, or by administering the wake-promoting drug modafinil (Petit et al., 2010). In the fly brain glycogen levels decrease after 3 h of SD but return to levels comparable to those measured during sleep after 6 h of SD (Zimmerman et al., 2004).

Since glycogen content critically depends on physical exercise and behavior, these variable findings may reflect true biological differences due to age and animal species. They may also depend on the specific brain region tested. Glycogen levels are heterogeneous in the cerebral tissue (Oe et al., 2016), with different regions showing different rates of glycogen depletion and production (Scharf et al., 2008), and wake-related increases in neuronal activity and aerobic glycolysis are not the same across the entire brain (Hobson and McCarley, 1971; Phelps, 1972; Goyal et al., 2014). On the other hand, inconsistent results may also reflect the technical challenge of measuring glycogen, which degrades rapidly in fresh tissue (Lowry et al., 1964; Fiala et al., 2003). An electron microscopy study showed that glycogen granules in astrocytes almost disappear in acute slices when compared with perfusion-fixed tissue (Fiala et al., 2003), suggesting that utilization of fixed tissue may lead to more consistent results.

Irrespective of the issues mentioned above, most studies so far have examined how sleep and wake affect glycogen levels across brain tissues indiscriminately, rather than around synapses, where energetically demanding processes take place (Attwell and Laughlin, 2001). Here, we employ serial-block face scanning electron microscopy (SB-SEM) to quantify glycogen content surrounding asymmetric (excitatory) synapses in layer II–III of the cerebral cortex of adolescent mice that were asleep for several hours or mostly awake for hours or days. We focused on the primary motor cortex, the same region where we had previously described sleep/wake related ultrastructural changes in the cell bodies of pyramidal neurons (de Vivo et al., 2016) and in synapses (de Vivo et al., 2017).

## Materials and Methods

### Animals

Homozygous B6.Cg-Tg(Thy1-YFP)16Jrs/J transgenic mice of either sex (4 week old) were used in this study. Sleep and synapse dynamics, including the analysis of perisynaptic astrocytic processes (PAPs), have been extensively characterized in this mouse strain (Maret et al., 2011; de Vivo et al., 2014, 2017; Bellesi et al., 2015, 2017). Mice were housed in groups (4 per cage) in environmentally controlled conditions (light/dark 12:12, light on at 8 am, 23 ± 1°C; food and water available *ad libitum* and replaced daily at 8 am). All animal procedures followed the National Institutes of Health Guide for the Care and Use of Laboratory Animals and facilities were reviewed and approved by the IACUC of the University of Wisconsin-Madison and were inspected and accredited by AAALAC. Mouse protocol number: M005697.

### Experimental Conditions and Protocols for Acute and Chronic Sleep Loss

**Figure 1:**
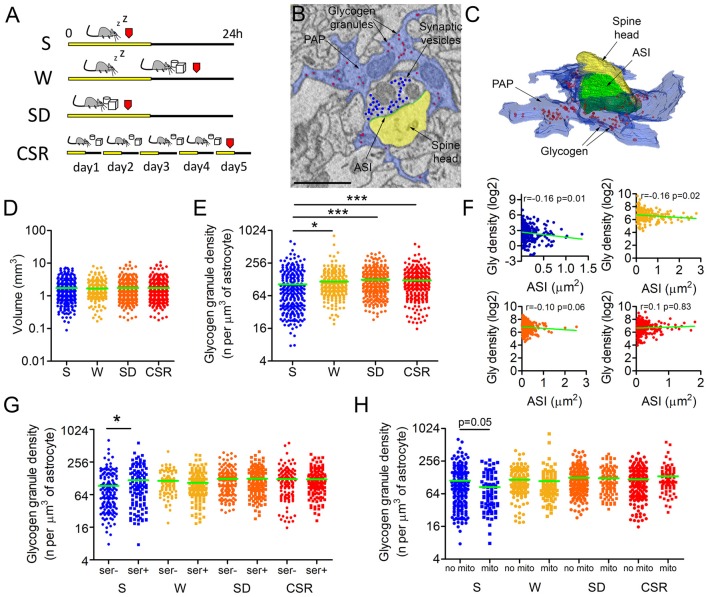
**(A)** Experimental design. Red marks indicate time of perfusion. **(B,C)** Representative example of a cortical synapse (layer II–III) surrounded by a peripheral astrocytic process (PAP, blue) and its 3D reconstruction **(C)**. Glycogen granules within the PAP are highlighted in red, axon-spine interface (ASI) is depicted in green, the spine head in yellow, and presynaptic vesicles in blue. **(D)** Regions of interest (ROIs) volumes across conditions (mean ± SEM; sleeping (S): 1.74 ± 0.08 mm^3^; spontaneously awake (W): 1.68 ± 0.07 mm^3^; sleep deprivation (SD): 1.75 ± 0.08 mm^3^; chronically sleep restricted (CSR): 1.75 ± 0.08 mm^3^) **(E)**. Glycogen granule density normalized by the astrocytic volume in S (mean ± SEM: 103.6 ± 5.4), W (116.3 ± 5.2), SD (125.7 ± 3.8) and CSR (123.1 ± 4.7) mice. *p < 0.05; ***p < 0.0001. **(F)** Correlation between the normalized glycogen (Gly) density expressed in log2 values and synaptic size, as measured by the ASI area for S, W, SD and CSR mice. **(G)** Glycogen granule density normalized by the astrocytic volume in synapses with or without (smooth endoplasmic reticulum, SER) at the postsynaptic level (mean ± SEM; S ser−: 92.9 ± 6.7 per μm^3^; S SER+: 118.2 ± 8.7 per μm^3^; W SER−: 115.1 ± 4.7 per μm^3^; W SER+: 105.9 ± 4.7 per μm^3^; SD SER−: 125.3 ± 5.6 per μm^3^; SD SER+: 126 ± 5.1 per μm^3^; CSR: SER−: 123.4 ± 7.4 per μm^3^; CSR SER+: 122.8 ± 5.7 per μm^3^). *p < 0.05. **(H)** Glycogen granule density normalized by the astrocytic volume in synapses with or without mitochondrion at the presynaptic level (mean ± SEM; S no mito: 111.8 ± 7.1 per μm^3^; S mito: 84.9 ± 6.9 per μm^3^; W no mito: 116.4 ± 5.2 per μm^3^; W mito: 109.4 ± 10.1 per μm^3^; SD no mito: 126.6 ± 4.7 per μm^3^; SD mito: 123.8 ± 6.5 per μm^3^; CSR: no mito: 117.8 ± 5.1 per μm^3^; CSR mito: 134.1 ± 9.7 per μm^3^). In **(D–G)** horizontal green lines indicate the mean.

Mice of the same age and comparable weight (~12 g at p25) were randomly assigned to four experimental groups (Figure 1A): (1) sleeping (S, *n* = 3) mice were killed during the light phase after 6–7 h of sleep and at the end of a long period of sleep (>45 min, interrupted by periods of wake of <4 min); (2) spontaneously awake (W, *n* = 3) mice were killed during the dark phase after ~7 h of wake and at the end of a long period of wake (>1 h, interrupted by periods of sleep of <5 min); (3) acutely SD (*n* = 3) mice were sacrificed during the light phase after 8 h of SD enforced by introducing novel objects and new bedding whenever the animals appeared drowsy. This method has been validated in previous studies using EEG-implanted mice (Bellesi et al., 2013, 2015) and leads to a reduction of total sleep >95% during the deprivation procedure; and (4) chronically sleep restricted (CSR; *n* = 3) mice were subjected to $4\frac{1}{2}$ days of chronic sleep restriction using a protocol optimized in our laboratory, consisting in exposure to novel objects during the day and forced locomotion on a slowly rotating treadmill during the night (see Bellesi et al., 2017 for details). In previous experiments we found that this method decreases overall sleep duration by ~70% (de Vivo et al., 2016). S, SD and CSR mice were killed at the same time of day (~4 pm), while W mice were killed at ~4 am (Figure 1A). To standardize the possible effects of physical exercise and environmental stressors on glycogen dynamics, during the dark period of the 2–4 days prior to the experiment both S and W mice had access to running wheels and were given 2–3 novel objects to explore.

### Video Recordings of Behavioral States

To avoid potential tissue damage and inflammation mice were not implanted with EEG electrodes. Behavioral states in S and W mice were estimated by quantifying motor activity using continuous video-monitoring with infrared cameras. As previously described (Maret et al., 2011), this method consistently estimates total sleep time with ≥90% accuracy, but it cannot differentiate NREM sleep from REM sleep. During the SD procedure SD and CSR mice were visually monitored by trained observers.

### Ultrastructural Studies

Quantification of glycogen content and analysis of synaptic ultrastructure were performed in the same image dataset previously used to characterize the dynamics of PAPs in S, SD, W and CSR mice (Bellesi et al., 2015), and a detailed description of the methods (staining, acquisition, and profiles segmentation) is reported there. Immediately before the perfusion, S, SD, W and CSR mice were quickly moved from the recording chamber to the perfusion room, they were picked by the tail, and gently positioned inside the anesthesia box prefilled with 3% isoflurane. After ~30 s, mice were moved on the perfusion tray equipped with an anesthesia mask and, when the corneal reflex and tail-pinch reflex were lost, the perfusion procedure began. All these procedures took a comparable amount of time (~1.5 min) in all mice and no signs of distress or pain were noticed. Under anesthesia mice (3 animals/group) were perfused intracardially with a solution of 0.05 M phosphate buffered saline (~5 s followed by 2.5% glutaraldehyde and 2% paraformaldehyde dissolved in 0.1 M sodium cacodylate buffer (41°C and pH 7.4, 250 ml in about 10 min at 300 mmHg) using a Perfusion One Instrument (Leica Biosystem). It is worth mentioning that the operators (MB and LV) performing the perfusions were not blind to the experimental condition. However, the procedure was consistently carried out in all mice using the same fixatives, and the time between the incision of the diaphragm and fixatives reaching the tissues was constantly <50 s. Brain tissue was sliced using a vibratome and stained with a solution of 1.5% potassium ferrocyanide/2% osmium tetroxide followed by 1% thiocarbonhydrazide, 2% osmium tetroxide and 1% uranyl acetate at 4°C. Next, the tissue was stained with a solution of lead aspartate, dehydrated and embedded with Durcopan resin and ACLAR film. Small volumes of tissue of about ~1 mm^3^ were obtained from frontal cortex (AP 1.85 mm; ML 1.5 mm), glued on the tip of a metal pin, and coated with silver paint to minimize specimen charging during imaging.

### Image Acquisition

Images were acquired using a SIGMA™ VP field emission scanning electron microscope (Carl Zeiss NTS Ltd.) equipped with 3View^®^ Technology (Gatan Inc.), and a backscattered electron detector (SBF-SEM). Image series were processed and analyzed using TrakEM2, a FIJI plug-in (Schindelin et al., 2012). Segmentation of spine heads, axon-spine interface (ASI), astrocytic profiles were performed manually by two operators blind to the experimental condition. Small cuboid regions of interest (ROI, 2–4 μm per side) of neuropil (layer II–III of primary motor cortex; 1.85 mm anterior to Bregma, 1.5 mm lateral) were selected around synapses randomly chosen in the neuropil. PAPs were recognized based on their distinctive shapes and the presence of glycogen granules, as well as the fact that they infiltrate among neuronal profiles and often contact parts of the synapse. ROIs did not include large dendrites or soma of neurons, glia or endothelial cells. For each ROI, astrocytic volume and ROI volume were estimated. Glycogen granules within the astrocytes were also manually annotated using the ball tool of TrakEM2. Annotation of the EM images was performed by two operators (MB and SK) blind to the experimental condition. Blinding procedure consisted in renaming file names using a computer-based shuffle key. The ball size was adjusted to cover the entire electron dense area occupied by the granule. Glycogen granules density was normalized to the astrocytic volume within each ROI. To estimate the number of glucose residues (GR) stored within glycogen granules, we applied the following formula: GR = 13*(2^∧^(diameter/(2*L)) − 1), where 13 is the average number of GR per chain, 2 is the branching degree, L is the length of one tier (approximately equal to 1.9 nm; Roach et al., 2012; DiNuzzo, 2013). Glycogen granules distance from ASI was computed by using a custom-made script in TrakEM2, which calculated the shortest path between a ball object and the nearest vertex point of ASI defined as a set of unique vertices of a meshed surface. Distance values were averaged within each ROI for subsequent analysis.

### Statistical Analysis

Non-parametric statistics were used for the analysis. Kruskal-Wallis test followed by Dunn’s test was used for multiple comparisons. Correlation analysis was performed with the Spearman test. Alpha was set to 0.05. No outliers were detected, and all the data points were included in the analysis.

## Results

### Wake Increases Glycogen Granule Density Around Cortical Synapses

To study the effects of sleep and wake on glycogen granule dynamics, we focused on cortical synapses of layers II/III of the mouse primary motor cortex. We manually drew cuboid ROI around randomly chosen axon-spine synapses in mice that were either sleeping (S, *n* = 308, ROIs from 3 mice), spontaneously awake (W, *n* = 268, 3 mice), acutely sleep deprived (SD; *n* = 339, 3 mice) or chronically sleep restricted (CSR, *n* = 339, 3 mice; Figure 1A). Within each ROI, the ASI of the synapse (the direct area of contact between the axonal bouton and the spine head), as well as the surrounding PAPs were manually segmented (Figures 1B,C). Astrocytic profiles were recognized due to their ability to interdigitate among neuronal structures, a clear cytoplasm, and the presence of numerous granules of glycogen (Figures 1B,C). PAP detection was facilitated by the ability to scroll within the stack along the third-dimension. A preliminary analysis of all stacks never revealed ultrastructural signatures of anoxia or necrosis (Van Reempts, 1984).

First, we confirmed that the amount of neuropil analyzed was comparable across experimental conditions (*p* = 0.64, Figure 1D). Then, we calculated the normalized density of glycogen granules, that is, all glycogen granules were annotated within each ROI and their number was divided by the volume of the astrocyte within the ROI. We found that the normalized density of glycogen granules was strongly affected by behavioral state (*p* < 0.0001), with W (*p* = 0.01), SD (*p* < 0.0001), and CSR (*p* < 0.0001) showing higher densities than S (Figure 1E). Moreover, the normalized density of glycogen granules was negatively correlated with the ASI in S (*r* = −0.16, *p* = 0.01) and W (*r* = −0.16, *p* = 0.02), indicating that after sleep and spontaneous wake small-medium synapses could count on higher number of glycogen granules than large synapses. However, this was no longer the case as the time spent awake increased: after SD the negative correlation was only a trend (*r* = −0.1, *p* = 0.06) and it disappeared after CSR (*r* = 0.1, *p* = 0.83), indicating that after extended wake glycogen granules were equally distributed to all synapses (Figure 1F). We recently demonstrated that synapses whose spine heads contain endoplasmic organelles (smooth endoplasmic reticulum, SER) are more likely to undergo sleep-related downscaling compared to synapses devoid of SER (de Vivo et al., 2017). Endoplasmic elements may mediate the recycling of membranes, glutamate receptors, and other proteins involved in activity-dependent structural changes (Park et al., 2006). Lactate, the by-product of glycogen breakdown, has been involved in synaptic plasticity (Gibbs et al., 2006; Suzuki et al., 2011). Thus, we also compared the density of glycogen granules across groups separately in synapses with and without SER (% of total synapses: S = 57.5; W = 35.9; SD = 47.2; CSR = 48.6). In both types of synapses wake groups had more granules than the sleep group, although the effect was stronger in synapses without SER (SER+: *p* = 0.01; SER−: *p* < 0.0001). We also assessed whether the presence of SER was related to the density of glycogen granules within each experimental group. This was the case in S mice (*p* = 0.016), while all wake conditions showed comparable (*p* > 0.05) densities of glycogen granules between SER+ and SER− synapses (Figure 1G).

Within the astrocytes, glycogen breakdown can ultimately lead to the production of lactate, which in turn can be extruded by these cells through dedicated lactate transporters (Bélanger et al., 2011). Outside the astrocytes, lactate acts as a signaling molecule (Yang et al., 2014) or can be up taken by neurons and used as an energetic substrate (Pellerin et al., 2007), particularly in case of intense synaptic activity (Brown and Ransom, 2015). Mitochondria are also a key source of energy at the synapse level (Ly and Verstreken, 2006), since they host the machinery to fully oxidize glucose and pyruvate derived from lactate dehydrogenation (Pellerin et al., 2007). Thus, we also compared the density of glycogen granules across groups separately in synapses with and without a presynaptic mitochondrion (% of total synapses: S = 69.5; W = 66.1; SD = 67.7; CSR = 59.9). We found that in both types of synapses the wake groups had more granules than the sleep group (Mito+: *p* < 0.0001; Mito−: *p* = 0.001). We also tested whether, within each experimental group, the presence of a presynaptic mitochondrion was related to the density of glycogen granules. In S mice the density of glycogen granules trended to be higher in synapses lacking the presynaptic mitochondrion (*p* = 0.05). However, this was not the case during waking: in all wake groups (W, SD, CSR) synapses with or without a mitochondrion showed a comparable density of glycogen granules (Figure 1H). This analysis was not applied to post-synaptic sites because mitochondria, albeit rarely present in the spine head (<1%), were almost always present along the dendrite within 2 μm from the point in which the spine neck emerged from the shaft.

### Size of Glycogen Granules Diminishes With Wake Periods of Increasing Duration

**Figure 2:**
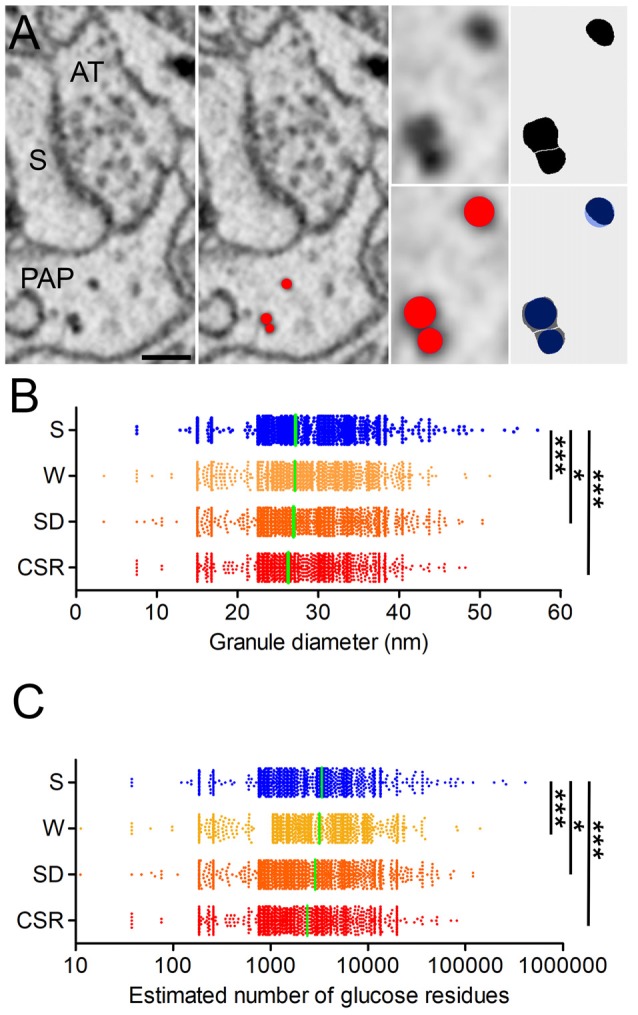
**(A)** Representative synapse surrounded by PAP. S, spine head; AT, axon terminal. Glycogen granules are segmented in red by the ball tool of TrakEM2. The ball can be manually adjusted in size to approximate the real shape of the granule, as showed by the high overlap between granules manually thresholded with FiJI (black) and the ones segmented using the ball tool (blue). Scale bar: 150 nm. **(B)** Granule diameter distribution in S (mean ± SEM; 27.2 ± 0.1) W (27.1 ± 0.1), SD (26.9 ± 0.1) and CSR (26.3 ± 0.1) mice. **(C)** Estimated number of glucose residues (GR) per granule, in S (mean ± SEM; 3364 ± 203), W (3,172 ± 94), SD (2,865 ± 68) and CSR (2,381 ± 54) mice. In **(B,C)** horizontal green lines indicate the mean. *p < 0.05; ***p < 0.0001.

Ultrastructural analysis in muscle cells has shown that the size of glycogen granules may vary depending on the metabolic state of the cell (Obel et al., 2012). Specifically, during recovery from prolonged exercise the diameter of the glycogen granule in muscle was negatively correlated with the rate of glycogen net synthesis, indicating that post-exercise restoration of glycogen occurs initially by an increase in granule number, followed by an increase in size (Marchand et al., 2007). The size of glycogen granules is comparable in astrocytes and muscle cells (Wender et al., 2000; Marchand et al., 2002), suggesting that the correlation between size of glycogen granules and rate of synthesis may also apply to the brain. Thus, we asked whether the wake-related increase in glycogen granule density was associated with changes in granule size. We measured the size of the granules using the ball object available in TrakEM2, which allowed us to approximate the size of the granule to a sphere (Figure 2A). Using this tool, we manually measured the size of all glycogen granules detected in S (*n* = 3,248), W (*n* = 3,718), SD (*n* = 5,399) and CSR (*n* = 4,289) mice. Quantitative analysis revealed an effect of behavioral state (*p* < 0.0001) and *post hoc* comparisons found that granule size was larger in S mice than in W (*p* < 0.0001), SD (*p* = 0.04), and CSR (*p* < 0.0001) animals. Differences in size were also found across wake groups, with greater granule size in W than in SD (*p* < 0.0001) and CSR (*p* < 0.0001) mice, and greater in SD than in CSR (*p* < 0.0001) mice (Figure 2B). Thus, glycogen granules progressively decrease in size with wake periods of increasing duration, likely reflecting increased turnover. Finally, since the size of the granules affects their metabolism (Roach et al., 2012; DiNuzzo, 2013) and we found that number and size of granules changed in opposite direction across sleep and wake, we estimated the number of glucose residues (GR) stored inside the glycogen granules as a measure of “cumulative glycogen,” to determine whether the wake groups still had more overall glycogen content than the sleep group (see formula in the “Materials and Methods” section). We found an overall effect of behavioral state (*p* < 0.0001) and pairwise comparisons showed that S had higher estimated number of GR than W (*p* < 0.0001), SD (*p* < 0.04), and CSR (*p* < 0.0001). Comparisons within the wake groups were also significant, with the number of GR progressively diminishing with time spent awake (W vs. SD, *p* < 0.0001; W vs. CSR, *p* < 0.0001; SD, vs. CSR, *p* < 0.0001, Figure 2C). Thus, short and long periods of wake are associated with an absolute decrease in glycogen granule size relative to sleep, likely reflecting increased turnover. On the other hand, sleep may favor glucose accumulation within the granules.

### Chronic Sleep Restriction Reduces the Distance of Glycogen Granules From the Synaptic Cleft

**Figure 3:**
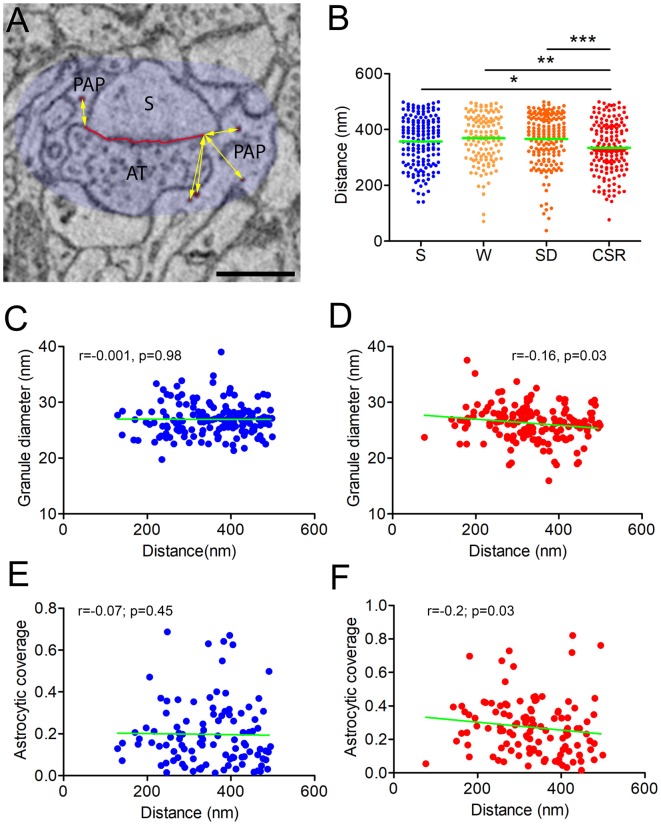
**(A)** Distance of the glycogen granules (red) was calculated within a region of 500 nm (here shadowed in light blue) from the nearest edge of the ASI (yellow arrows). Scale bar is 500 nm. **(B)** Distribution of the average distances in S (mean ± SEM; 357.2 ± 6.9), W (368.7 ± 7.6), SD (366.5 ± 6.9), and CSR (334.9 ± 6.1) mice. *p < 0.05; **p < 0.01; ***p < 0.0001. Horizontal green lines indicate the mean. **(C,D)** Correlation between the granule distance from the ASI and the granule diameter in S **(C)** and CSR **(D)** mice. **(E,F)** Correlation between the granule distance from the ASI and the astrocytic coverage of the synapse in S **(E)** and CSR **(F)** mice.

Our analysis on granule density and size indicates that wake promotes glycogen turnover, which may occur to cope with the greater energy demand at synapses imposed by the increased wake-related synaptic activity. Another mechanism through which wake could increase glycogen availability at synapses would be to move the glycogen granules closer to synapses, in order to deliver energy more rapidly. To verify this possibility, we measured the distance of every granule from the closest edge of the ASI. Since the overall distance might be affected by the ROI size, we considered only the granules that were within a range of 500 nm from the ASI (see Figure 3A). Statistical analysis revealed an effect of behavioral condition on the distance of the granules from synapses (*p* = 0.001). *Post hoc* comparisons confirmed that this distance was shorter in CSR mice relative to S (*p* = 0.03), W (*p* = 0.001) and SD (*p* = 0.0001) mice. All other pair-wise comparisons were not significant (Figure 3B).

Studies carried out in muscle have shown that small granules (also defined as progranules) are more subjected to turnover, whereas larger granules function as a reserve that can be used during high intensity exercise (Shearer and Graham, 2004). Thus, it is possible that, under more challenging conditions like during CSR, large granules are the ones that move closer to synapses to fulfill the energy requirements of synapses. To test if this was the case, we performed a correlation analysis between granule size and distance from the synapses in S and CSR ROIs. We did not find any significant correlation in S (*r* = −0.001, *p* = 0.98), suggesting small and large granules were uniformly distributed within the PAPs in this condition. By contrast, a modest but significant negative correlation was observed in CSR (*r* = −0.16, *p* = 0.03; Figures 3C,D). This indicates that in CSR the small glycogen granules are located farther from synapses, whereas large granules are in general closer to synapses.

Using the same data set of EM images of the current study, we previously found that the PAPs move closer to synapses after CSR (Bellesi et al., 2015). Thus, the reduced distance of the granules in CSR could be related to the increased astrocytic coverage around the synapses. We tested this possibility by computing a correlation analysis between the percentage of astrocytic coverage around the cleft, calculated as the portion of the ASI perimeter contacted by the PAP, and the average distance of the granules within each ROI in S and CSR. While in S this correlation was not significant (*r* = −0.07; *p* = 0.45), in CSR the astrocytic coverage negatively correlated with the average distance (*r* = −0.2; *p* = 0.03, Figures 3E,F), thus suggesting that the increased astrocytic coverage around the synapses can account, at least partially, for the diminished distance of the glycogen granules from the synapses in CSR.

Overall, these results indicate that distance of glycogen granules from synapses is reduced in CSR, with large granules being closer to synapses than smaller ones. This shortened distance is partially due to the increased astrocytic coverage of synapses after CSR.

## Discussion

We find that glycogen turnover in PAPs increases during wake while sleep promotes glycogen accumulation. Microwave irradiation and subsequent glycogen estimation on fresh tissue remain the gold standard procedures for assessing glycogen content (Oe et al., 2016), but they lack the spatial resolution to assess glycogen content specifically around the synapses. This is why in the current study we resorted to electron microscopy and used perfused tissue to maintain the ultrastructure. Perfusion allows good glycogen preservation compared to fresh live tissue, such as brain slices, where anoxia can deplete glycogen in a few minutes (Fiala et al., 2003). To minimize this problem we perfused the mice with a short flush of a warm prefixative solution (~5 s) followed by fixatives at 41°C. Importantly, in all mice no more than 50 s elapsed between the incision of the diaphragm and the time when fixatives reached the tissues, and all mice underwent the same steps (transfer to the perfusion room, anesthesia induction, perfusion, etc., see “Materials and Methods” section) within a comparable amount of time. The analysis of the ultrastructure never revealed manifest signs of anoxia or tissue necrosis in all the brain samples. Furthermore, our analysis is based on assessing relative differences among groups. Thus, even though some of the above described procedures (e.g., anesthesia) may affect brain metabolism (Boretius et al., 2013) and absolute glycogen content, it is unlikely that the changes in glycogen levels that we observe across groups are strongly biased by these procedures.

Due to the time-consuming nature of ultrastructural studies our analysis was restricted to the axo-spine synapses of the mouse primary motor cortex and specifically to layers II/III, because in this area ultrastructural synaptic changes due to learning and plasticity (Holtmaat and Svoboda, 2009) and to the sleep/wake cycle (de Vivo et al., 2017) are well documented. It remains to be seen whether these findings extend outside the small area that we analyzed. Aerobic glycolysis differs across brain regions (Goyal et al., 2014). Moreover, astrocytes in superficial and deep cortical layers differ in morphology and gene expression patterns (Lanjakornsiripan et al., 2018) and glycogen content may change across brain regions and cortical layers (Oe et al., 2016). Finally, our analysis did not consider the astrocytic processes close to dendrites and axons, whose glycogen content could also be affected by sleep and wake.

It is well established that glycogen breakdown follows the release of noradrenaline, serotonin, VIP and other neurotransmitters (Magistretti et al., 1993), which is typically higher in wake than in sleep. Therefore, one should expect increased glycogen consumption during wake. On the other hand, several studies have also shown that wake stimulates the production of glycogen, as indicated by the strong upregulation of the messenger RNAs coding for the protein targeting glycogen (PTG; Petit et al., 2002, 2010; Bellesi et al., 2015), a key enzyme of the metabolic pathway orchestrating glycogen synthesis, and for glycogenin (Petit et al., 2010), a protein leading the synthesis of new glycogen granules. Moreover, a nuclear magnetic resonance spectroscopy study found that rats kept awake with sensory stimuli for 5 h at the beginning of the light phase showed augmented incorporation of [1-(13)C]-labeled glucose into glycogen relative to rats that were left undisturbed (Morgenthaler et al., 2009). Glycogen consumption and synthesis work at different time scales. Glycogen breakdown is a very rapid process controlled by the enzyme glycogen phosphorylase, and does not require ATP (Brown and Ransom, 2015). By contrast, since glucose must be phosphorylated by a hexokynase to be used for glycogen formation, the buildup of glycogen is a relatively slow reaction and requires ATP (Hertz and Dienel, 2002). Thus, rapid glycogen consumption takes place following rapid shifts in neuronal activity, such as upon awakening, while glycogen production starts and proceeds slowly on the background, likely leading to the coexistence of both glycogen breakdown and glycogen formation during wake. Consistent with this interpretation, we found that the size of glycogen granules diminishes progressively with the duration of wake, strongly suggesting that glycogen turnover increases with the time spent awake.

We also found that the distance of glycogen granules from the synaptic cleft is specifically reduced after several days of substantial sleep restriction (to ~30% of baseline sleep). Whether glycogen granules can move within the astrocytes is currently unknown, but it is well established that astrocytic peripheral processes are extremely motile (Bernardinelli et al., 2014). In a previous study, we found that a few hours of wake are enough to move these processes closer to synapses (Bellesi et al., 2015). Moreover, we demonstrated that chronic sleep restriction induces an expansion of these processes nearby synapses, thus leading to increased astrocytic coverage of the spine head and synaptic cleft (Bellesi et al., 2015). Here, we found that in CSR mice the astrocytic coverage of synapses is negatively correlated with the distance of glycogen granules from the synaptic cleft. Moreover, we found that among all granules, the larger ones were those closer to the synapse after chronic sleep loss. In the brain, the turnover of glycogen particles occurs in the larger granules (outermost tiers), because they contain substantial amounts of glucose that can be rapidly mobilized (Roach et al., 2012; DiNuzzo, 2013). Recycling vesicles and reestablishing negative membrane potentials are extremely costly processes for the synapse (Attwell and Laughlin, 2001), and chronic sleep loss may require a sustained metabolic effort to prevent synaptic damage and failure (Bellesi et al., 2017). Thus, large granules nearby the synapses could provide the readily expandable energy required to meet the increased needs during sustained wake.

Glucocorticoid stress hormones are known to affect cerebral metabolism and, specifically, glucose uptake and utilization (Landgraf et al., 1978; Horner et al., 1990; Allaman et al., 2004). *in vitro* studies found that glycogen content in astrocytes is reduced in the presence of glucocorticoids (Tombaugh et al., 1992; Allaman et al., 2004) and increases after adrenalectomy (Passonneau et al., 1971). Glycogen levels in the rat cerebral cortex did not change after 6 h of sleep loss, but increased by more than 40% in the cerebral cortex of adrenalectomized rats (Gip et al., 2002). Thus, there is converging evidence that glucocorticoids prevent glycogen accumulation and we cannot rule out that the wake-related increase in glycogen turnover was due to increased levels of circulating corticosteroids. We note, however, that we recently found that relative to sleep, corticosterone plasma levels significantly increased in mice after short periods of enforced wake but showed only a trend to increase after chronic sleep restriction (Bellesi et al., 2018), while here we found that glycogen turnover was higher in chronic sleep restriction than in short SD.

In their original hypothesis, Benington and Heller (1995) proposed that depletion of glycogen during wake would lead to the progressive accumulation of adenosine, creating a homeostatic signal that reflects the buildup of sleep pressure over the course of wake. By focusing on glycogen content close to the synapses, we find that several hours of spontaneous or forced wake, as well as chronic sleep restriction, lead to increased glycogen turnover and an absolute reduction in the estimated number of glucose residues within the granules, while sleep promotes glycogen accumulation. In addition, by using electron microscopy we also find that some specific ultrastructural features of glycogen, the size of granules and their distance from the synaptic cleft, change according to wake duration, and may therefore reflect to some extent sleep pressure. Previous findings in our lab are also consistent with this conclusion. Specifically, in a recent study aimed at characterizing sleep/wake changes in the astrocytic transcriptome we found that *Ppp1r3c*, the gene coding for PTG, was strongly upregulated after both spontaneous wake and short SD relative to sleep (Bellesi et al., 2015). When the two wake conditions were compared to each other *Ppp1r3c* expression was also significantly higher after SD than after spontaneous wake (*p* = 0.03; W raw expression: 10.2 ± 0.17, 4 h SD raw expression: 10.6 ± 0.35; Bellesi et al., 2015), suggesting that PTG-related enzymatic activity increases with the duration of wake. These molecular changes, together with the current ultrastructural findings, suggest that glycogen turnover may to some extent reflect the accumulation of sleep pressure during wake.

Besides size, granule shape could be similarly important. Abnormal shape could reflect alterations of the granule structure, such as an inhomogeneity of chain growth owing to aberrant glycogen branching, which can lead to formation of insoluble aggregates (Roach et al., 2012; DiNuzzo et al., 2015). Although these modifications often characterize pathological states (e.g., Lafora disease), it is possible that chronic sleep restriction may also lead to alterations in granule structure. In our study, we used a semi-automatic method to segment glycogen granules, consisting in approximating the quasi-spherical shape of the granule to a sphere. With this method we could reliably estimate the granule size of thousands of granules, but we could not detect deviations in granule eccentricity or other shape abnormalities of the granules. More sophisticated methods of segmentation will be required in the future to test this possibility.

## Author Contributions

MB designed and performed the experiments, analyzed the data, and wrote the manuscript. LV performed the experiments and carried out the acquisition of the EM images. SK analyzed the EM images. GT and CC designed the experiments and wrote the manuscript.

## Conflict of Interest Statement

The authors declare that the research was conducted in the absence of any commercial or financial relationships that could be construed as a potential conflict of interest.
